# The Abundance of Plasmid-Mediated Quinolone Resistance Genes in *Enterobacter cloacae* Strains Isolated from Clinical Specimens in Kermanshah, Iran

**DOI:** 10.1155/2024/8849097

**Published:** 2024-04-08

**Authors:** Mandana Afsharian, Somayeh Asadi, Camellia Danesh, Reza Sedighi, Kohsar Karimi, Nooshin Miladi, Ronak Miladi, Mohsen Azizi, Nahid Madadi-Goli, Kamal Ahmadi, Mohammad Hossein Zamanian

**Affiliations:** ^1^Department of Infectious Disease, School of Medicine, Kermanshah University of Medical Sciences, Kermanshah, Iran; ^2^Student Research Committee, Kermanshah University of Medical Sciences, Kermanshah, Iran; ^3^Student Research Committee, Kurdistan University of Medical Sciences, Sanandaj, Iran; ^4^Department of Pediatrics, School of Medicine, Kermanshah University of Medical Sciences, Kermanshah, Iran; ^5^Department of Microbiology, School of Medicine, Kermanshah University of Medical Sciences, Kermanshah, Iran; ^6^Department of Mycobacteriology and Pulmonary Research, Pasteur Institute of Iran, Tehran, Iran

## Abstract

**Background:**

*Enterobacter cloacae* (*E. cloacae*) is one of the most common *Enterobacteriaceae* causing nosocomial infections. Plasmid-mediated quinolone resistance (PMQR) determinants have been considered recently. This study evaluated the abundance of PMQR genes in strains of *E. cloacae* obtained from clinical samples in Kermanshah, Iran.

**Methods:**

In this descriptive cross-sectional study, after collecting 113 isolates of *E. cloacae*, their identity was confirmed using specific biochemical tests. After determining their drug resistance patterns using disc diffusion, the phenotypic frequency of extended-spectrum beta-lactamase (ESBL)-producing isolates was measured by the double-disk synergy test (DDST) method. The isolates were examined for the presence of *qnrA*, *qnrB*, *qnrS*, and *aac(6′)-Ib-cr* genes by the polymerase chain reaction (PCR) assay.

**Results:**

The antibiotic resistance rate of *E. cloacae* isolates varied from 9.7% to 60.2%; among them, 78% were multidrug-resistant (MDR). The highest quinolone resistance was observed in ESBL-producing strains of *E. cloacae*. The frequency of positive isolates for PMQR and ESBL was 79.6% and 57.5%, respectively. The genes *aac(6′)-ib-cr* (70.8%) and *qnrB* (38.1%) had the highest frequency among other genes. The number of isolates simultaneously carrying 2 and 3 genes was 64 and 5 isolates, respectively.

**Conclusion:**

The obtained results indicate a high degree of quinolone resistance among ESBL-producing *E. cloacae* strains. Nevertheless, there was a significant relationship between the PMQR gene and ESBL-positive isolates. Therefore, special attention should be paid to molecular epidemiological studies on antibiotic resistance to quinolones and beta-lactamases in these strains.

## 1. Introduction

*Enterobacter* is one of the causes of nosocomial infections and belongs to the family Enterobacteriaceae. It exists as saprophytes everywhere and is part of the natural flora in the human intestine [1]. *E. cloacae* and *Enterobacter aerogenes* have been identified as major nosocomial pathogens among these bacterial species. *E. cloacae* is responsible for over 70% of these bacterial infections [2]. Due to various virulence factors, such as biofilm-forming ability, toxins, cytotoxicity, and hemolysin release, this bacterium can lead to several nosocomial infections, including pneumonia, urinary tract infections, surgical wounds, skin and soft tissue infections, and bacteremia [3, 4]. Inappropriate antibiotic prescribing, overuse, and the underdevelopment of new antibiotics have rapidly caused antibiotic-resistant bacteria to emerge, causing nearly 700,000 deaths annually [5]. Many broad-spectrum antibiotics are used to treat bacterial infections, and their improper use has led to widespread resistance, reducing the effectiveness of these antibiotics. Recent studies have reported cases of increased drug resistance in *Enterobacter* strains and the emergence of multidrug-resistant (MDR) strains of this bacterium [6, 7]. *Enterobacter* strains are often associated with the MDR phenotype due to their ability to acquire mobile genetic factors containing resistance genes and adaptability to the hospital environment. These bacteria are intrinsically resistant to ampicillin, amoxicillin, first-generation cephalosporins, and cefoxitin due to the expression of AmpC beta-lactamase. Additionally, extended-spectrum beta-lactamase (ESBL) production in these bacteria makes them challenging to treat [8]. Quinolones and fluoroquinolones are highly applied for the treatment of various bacterial infections. Resistance to quinolones can be caused by chromosomal mutations in bacterial genes encoding quinolone target proteins, mutations that cause decreased drug accumulation due to decreased uptake or increased efflux, or plasmid-localized genes associated with quinolone resistance [9, 10]. Three classes of plasmid-mediated quinolone resistance (PMQR) genes have been identified based on their mode of action, including *qnr* proteins, the *aac(6′)-Ib-cr* genes, and efflux pump genes *oqxA*, *oqxB*, and *qepA* [11]. The *qnr* genes are one of the major PMQR factors, increasing drug resistance in bacteria due to their location on genetic factors [12]. In addition to inducing quinolone resistance, PMQR might play an important role in resistance to other antibiotics, particularly aminoglycosides and beta-lactamases [13]. Currently, seven groups of *qnr* genes have been identified, including *qnrA*, *qnrB*, *qnrS*, *qnrC*, *qnrD*, and *qnrVC* [14]. The *qnr* gene induces quinolone resistance by blocking deoxyribonucleic acid (DNA) gyrase and topoisomerase IV. Quinolone resistance is also induced by an aminoglycoside acetyltransferase called *aac(6)-ib-cr*, which reduces sensitivity to ciprofloxacin. Additionally, the *aac(6)-ib-cr* gene, besides causing aminoglycoside resistance, confers resistance to fluoroquinolones [15]. Quinolones are the most important antibiotics used in the treatment of various bacterial infections. Unfortunately, PMQR-mediated resistance has been widely reported in *Enterobacteriaceae* worldwide in recent decades. On the other hand, the presence of PMQR determinants can increase QRDR mutations, which increases fluoroquinolone resistance [16]. As a result, determining the frequency of PMQR genes in different species of *Enterobacteriaceae* can provide important information to determine the epidemiology and understand the abundance and distribution of PMQRs to prevent the irrational use of these antibiotics and the spread of drug resistance. Considering that there is no study on the frequency of PMQR genes in *Enterobacter* strains isolated in this province, the present study evaluated the frequency of PMQR genes in *E. cloacae* strains collected from patient samples in Kermanshah City, Iran.

## 2. Materials and Methods

### 2.1. Bacterial Isolates

In this cross-sectional study conducted from February 2020 to January 2021, we prepared 113 nonreplicating clinical isolates of *E. cloacae* from patients referred to Imam Reza Hospital in Kermanshah. Clinical samples included urine, wound, blood, trachea, sputum, cerebrospinal fluid (CSF), bronchoalveolar lavage (BAL), and catheter specimens. This study focused exclusively on *E. cloacae* isolates from the clinical samples of hospitalized patients, excluding other *Enterobacter* species and environmental samples. The present research was approved by the Ethics Committee of Kermanshah University of Medical Sciences (No. IR.KUMS.REC.1399.754), and informed consent was obtained from all patients. Initially, the collected specimens were cultured on eosin methylene blue agar (EMB) and McConkey agar (Merck, Germany) under sterile conditions. Subsequently, specific tests, including culture in IMVIC and triple sugar iron (TSI) agar medium, Simmons citrate agar (HiMedia Co., India), and indole test, were employed to identify *E. cloacae*. The *E. cloacae* samples identified in tryptic soy broth (TSB) were then preserved with 15% glycerol at −70°C.

### 2.2. Antimicrobial Susceptibility Assessment

Antibiotic sensitivity testing of the isolates was conducted following Clinical and Laboratory Standards Institute (CLSI) guidelines, using 17 antibiotic discs (MAST, UK) that included ciprofloxacin (5 *μ*g), nalidixic acid (30 *μ*g), norfloxacin (10 *μ*g), gatifloxacin (5 *μ*g), levofloxacin (5 *μ*g), ceftazidime (30 *μ*g), ofloxacin (5 *μ*g), cefotaxime (30 *μ*g), aztreonam (30 *μ*g), ceftriaxone (30 *μ*g), imipenem (10 *μ*g), nitrofurantoin (30 *μ*g), colistin (10 *μ*g), chloramphenicol (10 *μ*g), gentamicin (10 *μ*g), tobramycin (10 *μ*g), and amikacin (30 *μ*g). The Kirby–Bauer method was used for this test, with the standard concentration of McFarland 0.5 (1.5 × 10^8^) of bacteria applied for antimicrobial sensitivity testing. *Escherichia coli* strain ATCC 25922 served as a control for the antibiogram test, and isolates resistant to three or more types of antibiotics were classified as MDR *E. cloacae* strains.

### 2.3. Extended-Spectrum Beta-Lactamases Detection

The isolates characterized by minimum inhibition zone diameters of 22, 25, and 27 mm for ceftazidime, ceftriaxone, and cefotaxime, respectively, were evaluated for ESBL genes. To confirm ESBL production, the combined disk (CD) approach was used, employing 30 *μ*g ceftazidime and cefotaxime disks impregnated with 10 *μ*g clavulanic acid (MAST, UK) on Mueller–Hinton agar (HiMedia Co., India), following the disk diffusion method. After 24 hours of incubation at 37°C, strains with a minimum inhibition zone diameter of ≥5 mm compared to a single disc of the same antibiotic were considered ESBL producers.

### 2.4. Detection of PMQR Genes

The isolates' genomes were extracted through boiling, and the frequency of the target genes (Supplementary file (available here)) was determined using their specific primers (Tekapo Biot Company, Iran) via polymerase chain reaction (PCR) [12, 17]. The level of extracted DNA specimens was measured at 260 nm using a Nanodrop Synergy HTX (BioTek Instrument, Inc. USA), resulting in a concentration of 34 pmol/uL. The purity of the DNA extracted at 280/260 nm wavelength was 1.82. The PCR reaction, with a volume of 25 *μ*L, included Master Mix (12.5 *μ*L) (Sinocloon Company, Iran), 1 *μ*L of primer, bacterial DNA (2 *μ*L), and sterilized distilled water up to 25 *μ*L. The PCR procedure comprised initial denaturation (94°C/5 min), 35 basic cycles, and extension (10 min/72°C). In this study, in addition to using *E. coli* J53 strains containing pMG252, pMG298, and pMG306 as positive controls for *qnrA*, *qnrB*, and *qnrS* genes, respectively, we also used isolates carrying quinolone resistance genes from a previous study. The final products were detected through electrophoresis on 1% agarose gel (70 V, 1 h) with ethidium bromide (0.5 *μ*g/mL) in the Tris-EDTA buffer. An ultraviolet illuminator (ProteinSimple, USA) was employed to observe the gel, with distilled water serving as a negative control for each PCR.

### 2.5. Statistical Analysis

The data were analyzed using SPSS software (version 20) with the chi-square and Fisher's exact tests. A *P* value less than 0.05 was considered significant.

## 3. Results

**Figure 1:**
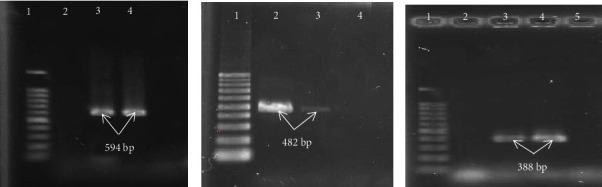
PCR product electrophoresis of PMQR genes. (a) Lane 1: 100 bp DNA ladder; lane 2: negative control; 3: positive control (594 bp); 4: positive sample (594 bp). (b) Lane 1: 100 bp DNA ladder; 2: positive control (482 bp); 3: positive sample (482 bp); lane 4: negative control. (c) Lane 1: 100 bp DNA ladder; lane 2: negative control; 3: positive control (388 bp); 4: positive sample (388 bp).

A total of 113 clinical strains of *E. cloacae* were collected from 113 patients, including 47 (41.6%) male and 66 (58.4%) female subjects, with a mean age of 36.42 ± 11.59 years, ranging from 16 to 72 years. These strains were obtained from patients at Imam Reza Hospital in Kermanshah, Iran. The highest and lowest frequencies of *E. cloacae* isolates were found in urine samples (43 : 38.1%) and BAL samples (5 : 4.4%), respectively. Furthermore, most isolates were obtained from the urology, intensive care unit (ICU), and outpatient wards (Table 1). The results of the antibiotic resistance pattern of *E. cloacae* indicated that the highest antibiotic resistance was observed for nalidixic acid (68 : 60.2%) and ciprofloxacin (66 : 58.4%); however, the highest sensitivity was found for colistin (11 : 9.7%). The frequency of MDR strains was 78% (89 isolates). Among the 113 isolates, the frequency of quinolone-resistant isolates was determined to be 53.7%, with the highest and lowest levels of resistance to nalidixic acid (60.2%) and gatifloxacin (33.6%), respectively. Notably, quinolone-resistant isolates exhibited significantly higher drug resistance than quinolone-sensitive isolates, particularly against cephalosporins and aminoglycosides (Table 2). Based on the results of the double-disk synergy test (DDST) method, the frequencies of positive and negative ESBL isolates were determined to be 65 (57.5%) and 48 (42.5%), respectively. A high degree of resistance to all tested quinolones was observed in ESBL-producing isolates compared to non-ESBL-producing isolates. Statistically, there was a significant relationship between the drug resistance properties of ESBL-positive and ESBL-negative strains (Table 3). The frequency of PMQR in the 113 *E. cloacae* isolates was 77% (87 cases), and among these strains positive for ESBL, it was 93.8% (61/65 cases). The highest frequency of quinolone resistance genes was identified for the *aac(6′)-Ib-cr* gene (80 : 70.8%). The frequencies of the remaining genes were 20 (17.7%) and 43 (38.1%) for *qnrS* and *qnrB* genes, respectively. None of the strains possessed the *qnrA* gene. The frequency of the *qnrB* gene was the highest in the age group of 16–30 years (14/19 cases: 73.7%), which was statistically significant. However, in other cases, there was no significant relationship between the PMQR gene frequency and patient gender or different age groups (*P* > 0.05). The frequencies of isolates with two concurrent genes included 35.4%, 4.4%, and 13.8% for *qnrB* + *aac(6′)-Ib-cr*, *qnrB* + *qnrS*, and *qnrS* + *aac(6′)-Ib-cr*, respectively. In addition, 5 isolates simultaneously possessed 3 genes: *qnrB* + *qnrS* + *aac(6′)-Ib-cr*. The highest frequency of ESBL- and PMQR-positive strains was related to *E. cloacae* isolates obtained from clinical urine and blood samples in the urology and hospitalized wards (Table 2). From a statistical point of view, there was a significant relationship between the presence of the studied resistance genes and the drug resistance patterns (*P* < 0.05) (Supplementary file (available here)). The majority of the genes studied were detected in the ESBL strains, and in some cases, this relationship was statistically significant (Table 4).  Figure 1 shows the PCR results for the *qnrB*, *qnrS*, and *aac(6′)-Ib-cr* genes.

## 4. Discussion

In recent years, there has been a global increase in infections caused by MDR *E. cloaca*e strains [18]. The highest frequency of identified strains was isolated from urine and wound samples. Previous studies have also reported isolating many strains of these bacteria from urine samples [2, 5, 19]. However, Liu et al. reported the highest frequency of *E. cloacae* isolation from sputum samples [20]. Among the studied strains, the highest resistance was observed against nalidixic acid (60.2%), ciprofloxacin, and tobramycin (58.4%). The overall resistance rates to quinolones, cephalosporins, and aminoglycosides were determined to be 53.7%, 50.4%, and 49.2%, respectively. Of 113 *E. cloacae* isolates, 89 (78%) exhibited multidrug resistance. The frequency of MDR *E. cloacae* isolates has been reported to range from 69.9% to 75% in other studies [3, 7, 21]. In Uzunović et al.'s study, over 66% and 28% of *E. cloacae* isolates were resistant to cephalosporins and aminoglycosides, respectively [22]. In 2022, the resistance rates to cephalosporins, gentamicin, ciprofloxacin, and imipenem in these isolates were 100%, 82.3%, 60.8%, and 7.5%, respectively [23]. Colistin (9.7%) and imipenem (14.2%) exhibited the lowest drug resistance rates among the isolates in this study. Other studies have reported lower antibiotic resistance rates in *E. cloacae* isolates against imipenem and colistin [20–23]. However, in Ebomah and Okoh survey, more than 70% of these bacterial strains were resistant to carbapenems [24]. Differences in resistance outcomes across studies might be attributed to variations in bacterial populations, their prevalence in hospital settings, the distribution of resistance genes, variations in antibiotic use patterns, and patient management practices. The frequency of ESBL production among *E. cloacae* strains in this study was determined to be 57.5%. The ESBL-positive isolates of this bacterium have been reported at rates ranging from 59.1% to 100% in other studies [6, 7, 25]. In ESBL-producing isolates, higher drug resistance was observed for all tested quinolones (*P* < 0.05). Azargun et al. reported a significant relationship between ESBL activity and fluoroquinolone resistance [26]. Discrepancies in these findings might be due to prolonged hospital stays and inappropriate and increased antibiotic use.

The frequency of PMQR in *E. cloacae* strains and ESBL-positive strains in this study was 77% and 93.8%, respectively. The high prevalence of PMQR and ESBL genes in this study can be indicative of the indiscriminate use of various antibiotics, including quinolones and beta-lactamases, followed by the spread of antibiotic resistance among bacterial isolates, which was consistent with the results of the drug resistance patterns. The highest frequency of the *qnr* gene was found in *E. cloacae* strains related to the *aac(6′)-Ib-cr* gene (70.8%). Given the location of the *qnr* gene in plasmids containing many resistant genes, including beta-lactamases, the higher frequency of quinolone-resistant isolates in ESBL-producing *E. cloacae* strains in the present study might be reasonable. Markovska et al. reported a PMQR gene frequency of 59% in *Enterobacter* strains [27]. In Azargon et al.'s study, similar to the results of the present study, the highest PMQR gene frequencies were found in ESBL-producing isolates. They also observed a significant correlation between the PMQR gene frequency and ESBL-positive isolates, which is consistent with the results of the present study [26]. In another study performed in 2020, the highest PMQR gene frequency among ESBL-producing *Klebsiella pneumoniae* strains was linked to *aac(6′)-Ib-cr* (55.6%) and *qnrB* (34.9%) [12]. The aforementioned results suggest a high frequency of quinolone-resistant genes, the presence of resistant bacterial strains, and the widespread prevalence of resistant genes among them. In Huang et al.'s study, 68.8% of *E. cloacae* isolates had the PMQR gene. They reported that the highest frequency of PMQR-positive and *aac(6′)-Ib-cr*-positive isolates was among the ESBL-producing isolates of this bacterium, which is consistent with the results of the current study [25]. Previous studies have shown that the presence of the PMQR gene is significantly associated with other antibiotic-resistant genes, including the ESBL gene, in bacterial isolates [28]. The present study's most common *qnr* gene was *qnrB* (38.1%). Other studies have also reported *qnrB* as the most frequently detected *qnr* gene, similar to the findings of the current study. For instance, in Bolourchi et al.'s study, 3 of 4 isolates of *E. cloacae* carried this gene [29]. Markovska et al. reported the frequency of *qnrB* as 90% [27]. Among 113 strains of *E. cloacae*, the frequency of *qnrS* was determined to be 17.7%. In other studies, the frequency of this gene was reported as 24.1% and 37.1%, respectively [19, 25]. In Guillard et al.'s study, of the 31 PMQR-harboring *E. cloacae* isolates, 13 (42%) carried *qnr* only, and 17 (55%) carried both *qnr* and *aac(6′)-Ib-cr* [30]. In another study, the frequency of *E. cloacae* isolates carrying the *qnr* gene was reported at 60.3%, with *qnrB1* being the most common (38.8%), followed by *qnrS1* (24.1%). None of the isolates of the present study contained *qnrA*, which is consistent with studies conducted in Iran [10, 19]. However, in a study conducted in China, all 4 strains of *E. cloacae* had this gene [25]. In the current study, only 4.4% of isolates simultaneously possessed both *qnrB* and *qnrS* genes. In Peymani et al.'s study, there was a higher frequency (8.6%) of these isolates containing these genes together [19]. This is the first study on the prevalence of quinolone resistance mediated by the plasmid *aac(60)-Ib-cr* in *E. cloacae* in Kermanshah City. According to the results of a previous study and the findings of this study, it was shown that the frequency of the *aac(6′)-Ib-cr* gene was higher than that of the *qnr* gene in isolated *Enterobacteriaceae* strains. The main limitations of this study were the lack of research on other quinolone resistance mechanisms and the lack of plasmid DNA analysis.

## 5. Conclusions

In the present study, a high level of antibiotic resistance was observed in *E. cloacae* strains capable of producing PMQR and ESBL. This resistance can be attributed to the presence of plasmids in ESBL-producing isolates, which often carry various resistance genes. Considering the significant prevalence of the PMQR gene in ESBL-producing isolates among the samples in the current study, it is essential to emphasize the rational and appropriate use of various fluoroquinolones in the treatment of bacterial infections. The dissemination of PMQR and ESBL gene-containing plasmids is a cause for concern, as it can facilitate the selection and proliferation of MDR strains within the community. Therefore, conducting regular and comprehensive studies to gather more information about isolated *E. cloacae* strains in this context is imperative.

## Figures and Tables

**Table 1:** Distribution of PMQR gene and ESBL-positive *E. cloacae* strains by sex, age group, specimens, and wards.

	ESBL (No. 65)	*qnrB* (No. 43)	*qnrS* (No. 20)	*aac(6′)-Ib-cr* (No. 80)
Gender				
Male	27	12	12	33
Female	38	31	8	47
Age (year)				
16–30	14	10	3	15
31–45	22	14	10	26
46–60	20	12	4	27
61–72	9	7	3	12
Specimens				
Urine	23	15	7	33
Wound	8	7	3	12
Blood	15	12	3	18
Trachea	9	5	4	6
Sputum	2	1	0	2
CSF	4	1	2	4
BAL	4	2	1	5
Wards				
Urology	22	18	7	34
ICU	14	7	4	13
Surgery	1	1	0	2
Burn	5	3	1	6
Gastrointestinal	7	4	3	7
Outpatient	16	10	5	18

**Table 2:** Antibiotic resistance rates of quinolone-resistant and quinolone-susceptible in *E. cloacae* isolates.

Antibiotic	Total resistance *n* (%)			Quinolone-resistant *n*: 87 (77%)			Quinolone-susceptible *n*: 26 (23%)			*P*-value
	R	I	S	R	I	S	R	I	S	
CTX	54 (47.8)	0	59 (52.2)	53 (60.9)	0	34 (39.1)	4 (15.4)	0	22 (84.6)	0.001
CAZ	51 (45.1)	0	62 (54.9)	50 (57.5)	2 (2.3)	35 (40.2)	3 (11.5)	0	23 (88.5)	0.001
CRO	63 (55.8)	0	50 (44.2)	57 (65.5)	0	30 (34.5)	6 (23.1)	0	20 (76.9)	0.001
ATM	53 (46.9)	2 (1.8)	58 (51.3)	48 (55.2)	0	39 (44.8)	3 (11.5)	0	23 (88.5)	0.001
GM	57 (50.4)	2 (1.8)	54 (47.8)	52 (59.8)	2 (2.3)	33 (37.9)	5 (19.2)	0	21 (80.8)	0.001
AK	44 (38.9)	2 (1.8)	67 (59.3)	41 (47.1)	2 (2.3)	44 (50.6)	3 (11.5)	0	23 (88.5)	0.001
TN	66 (58.4)	3 (2.7)	44 (38.9)	51 (58.6)	3 (3.5)	33 (37.9)	15 (57.7)	0	11 (42.3)	0.513
IMI	16 (14.2)	6 (5.3)	91 (80.5)	11 (12.6)	4 (4.6)	72 (82.8)	5 (19.2)	2 (7.7)	19 (73.1)	0.293
NI	34 (30.1)	0	79 (69.9)	25 (28.7)	0	62 (71.3)	9 (34.6)	0	17 (65.4)	0.365
CO	11 (9.7)	2 (1.8)	100 (88.5)	29 (33.3)	0	58 (66.7)	9 (34.6)	0	17 (65.4)	0.540
C	38 (33.6)	0	75 (66.4)	9 (10.3)	0	78 (89.7)	2 (7.7)	2 (7.7)	22 (84.6)	0.144

R: resistance; S: sensitive; I: intermediate; CTX: cefotaxime; CAZ: ceftazidime; CRO: ceftriaxone; ATM: aztreonam; GM: gentamicin; AK: amikacin; TN: tobramycin; IMI: imipenem; NI: nitrofurantoin; CO: colistin; C: chloramphenicol.

**Table 3:** Association between antibiotic resistance in ESBL-positive and ESBL-negative *E. cloacae* isolates.

Antibiotic	Total resistance no (%)			ESBL-positive 65 isolates			ESBL-negative 48 isolates			*P*-value
	R	I	S	R	I	S	R	I	S	
NA	68 (60.2)	4 (3.5)	41 (36.3)	49 (75.4)	2 (3.1)	14 (21.5)	19 (39.6)	2 (4.2)	27 (56.2)	0.001
CIP	66 (58.4)	8 (7.1)	39 (34.5)	47 (72.3)	5 (7.7)	13 (20)	19 (39.6)	3 (6.2)	26 (54.2)	0.001
NOR	56 (49.6)	8 (7.1)	49 (43.4)	39 (60)	4 (6.2)	22 (33.8)	17 (35.4)	4 (8.4)	27 (56.2)	0.83
LEV	45 (39.8)	3 (2.7)	65 (57.5)	36 (55.4)	0	29 (44.6)	8 (16.6)	4 (8.4)	36 (75)	0.75
GAT	38 (33.6)	0	75 (66.4)	30 (46.2)	0	35 (53.8)	8 (16.6)	0	40 (83.4)	0.001
OFX	49 (43.4)	4 (3.5)	60 (53.1)	35 (53.8)	3 (4.6)	27 (41.6)	13 (27.1)	2 (4.2)	33 (68.7)	0.013
CTX	57 (50.4)	0	56 (49.6)	51 (78.5)	0	14 (21.5)	6 (12.5)	0	42 (87.5)	0.001
CAZ	51 (45.1)	0	62 (54.9)	51 (78.5)	2 (3.1)	12 (18.4)	2 (4.2)	0	46 (95.8)	0.001
CRO	63 (55.8)	0	50 (44.2)	55 (84.6)	0	10 (15.4)	8 (16.6)	0	40 (83.4)	0.32
ATM	53 (46.9)	2 (1.8)	58 (51.3)	50 (76.9)	0	15 (23.1)	1 (2.1)	0	47 (97.9)	0.001
GM	57 (50.4)	2 (1.8)	54 (47.8)	52 (80)	1 (1.6)	12 (18.4)	5 (10.4)	1 (2.1)	42 (87.5)	0.001
AK	44 (38.9)	2 (1.8)	67 (59.3)	41 (63.1)	1 (1.6)	23 (35.3)	3 (6.2)	1 (2.1)	44 (91.7)	0.001
TN	66 (58.4)	3 (2.7)	44 (38.9)	38 (58.5)	3 (4.6)	24 (36.9)	28 (58.3)	0	20 (41.7)	0.358
IMI	16 (14.2)	6 (5.3)	91 (80.5)	10 (15.4)	3 (4.6)	52 (80)	6 (12.5)	3 (6.2)	39 (81.3)	0.270
NI	34 (30.1)	0	79 (69.9)	23 (35.4)	0	42 (64.6)	11 (22.9)	0	37 (77.1)	0.155
CO	11 (9.7)	2 (1.8)	100 (88.5)	9 (13.8)	0	56 (86.2)	2 (4.2)	2 (4.2)	44 (91.6)	0.156
C	38 (33.6)	0	75 (66.4)	24 (36.9)	0	41 (63.1)	14 (29.2)	0	34 (70.8)	0.290

R: resistance; S: sensitive; I: intermediate. NA: nalidixic acid; CIP: ciprofloxacin; NOR: norfloxacin; LEV: levofloxacin; GAT: gatifloxacin; OFX: ofloxacin; CTX: cefotaxime; CAZ: ceftazidime; CRO: ceftriaxone; ATM: aztreonam; GM: gentamicin; AK: amikacin; TN: tobramycin; IMI: imipenem; NI: nitrofurantoin; CO: colistin; C: chloramphenicol.

**Table 4:** The frequency of PMQR genes in positive and negative ESBL-producing *E. cloacae* isolates.

Genes	ESBL-positive	ESBL-negative	*P*-value
*qnrA*	—	—	—
*qnrB*	33	10	^*∗*^0.001
*qnrS*	14	6	0.160
*aac(6′)-Ib-cr*	59	21	^*∗*^0.001

^*∗*^Significant.

## Data Availability

All data supporting the results are contained in the manuscript.

## Supplementary Materials

Supplementary Materials The primers and temperature cycles used in the PCR reaction to check the genes studied in this study are listed in the table primers (supplementary file). From a statistical point of view, there was a significant relationship between the presence of the studied resistance genes and the drug resistance patterns (*P* < 0.05). Of these, the most statistically significant correlation was observed between the presence of the *aac(6′)-Ib-cr* gene and resistance to most of the antibiotics studied, including quinolones, cephalosporins, and aminoglycosides (supplementary file).
