# Supplementary material for: The Abundance of Plasmid-Mediated Quinolone Resistance Genes in Enterobacter cloacae Strains Isolated from Clinical Specimens in Kermanshah, Iran
Source: Can J Infect Dis Med Microbiol. 2024 Apr 8;2024:8849097. doi: 10.1155/2024/8849097 (PMC11018368; doi:10.1155/2024/8849097)
Supplement: Supplementary Materials — The primers and temperature cycles used in the PCR reaction to check the genes studied in this study are listed in the table primers (supplementary file). From a statistical point of view, there was a significant relationship between the presence of the studied resistance genes and the drug resistance patterns (P < 0.05). Of these, the most statistically significant correlation was observed between the presence of the aac(6′)-Ib-cr gene and resistance to most of the antibiotics studied, including quinolones, cephalosporins, and aminoglycosides (supplementary file). [file 8849097.f1.docx]

The primers and temperature cycles used in PCR reaction to check the genes studied in this study are listed in the table below.

| **Primers and temperature cycles used in PCR** | | | | | |
| --- | --- | --- | --- | --- | --- |
| 35 Cycles | | | | | |
| Primer | Sequence (5́-3) | Denaturation  94°C | Annealing  1 min | Extension  72°C | Product size (bp) |
| *qnrA* | TTCTCACGCCAGGATTTGAG TGCCAGGCACAGATCTTGAC | 1 min | 57°C | 1 min | 571 |
| *qnrB* | TGGCGAAAAAATTGAACAGAA GAGCAACGATCGCCTGGTAG | 1 min | 55°C | 1 min | 594 |
| *qnrS* | GACGTGCTAACTTGCGTGAT AACACCTCGACTTAAGTCTGA | 1 min | 54°C | 1 min | 388 |
| *aac (6′)-Ib-cr* | TTGCGATGCTCTATGAGTGGCTA CTCGAATGCCTGGCGTGTTT | 1 min | 57°C | 1 min | 482 |

From a statistical point of view, there was a significant relationship between the presence of the studied resistance genes and the drug resistance patterns (*P* <0.05). Of these, the most statistically significant correlation was observed between the presence of the *aac(6')-Ib-cr* gene and resistance to most of the antibiotics studied, including quinolones, cephalosporins, and aminoglycosides (Table below).

| **Association between resistance genes to quinolones and efflux pumps with drug resistance patterns *E.cloacae* isolates** | | | | | | | | | |
| --- | --- | --- | --- | --- | --- | --- | --- | --- | --- |
| Antibiotic | ***Qnr B***  **(N = 43)** | | | ***Qnr S***  **(N = 20)** | | | ***aac(6’)-Ib-cr***  **(N = 80)** | | |
|  | **R** | **I** | **S** | **R** | **I** | **S** | **R** | **I** | **S** |
| **NA** | ^🟋^42 | 1 | 0 | 15 | 0 | 5 | ^🟋^58 | 1 | 21 |
| **CIP** | ^🟋^41 | 2 | 0 | 15 | 2 | 3 | ^🟋^53 | 7 | 20 |
| **NOR** | 33 | 4 | 6 | 14 | 1 | 5 | ^🟋^45 | 7 | 28 |
| **LEV** | 29 | 2 | 12 | 10 | 0 | 10 | ^🟋^38 | 1 | 39 |
| **GAT** | 25 | 0 | 18 | 9 | 0 | 11 | ^🟋^33 | 0 | 47 |
| **OFX** | ^🟋^31 | 3 | 9 | 12 | 1 | 7 | ^🟋^39 | 5 | 36 |
| **CTX** | 29 | 0 | 14 | 11 | 0 | 9 | ^🟋^52 | 0 | 28 |
| **CAZ** | ^🟋^27 | 1 | 15 | 7 | 0 | 13 | ^🟋^42 | 2 | 29 |
| **CRO** | 26 | 0 | 17 | 13 | 0 | 7 | ^🟋^54 | 0 | 26 |
| **ATM** | 26 | 0 | 17 | 7 | 0 | 13 | ^🟋^47 | 0 | 33 |
| **GM** | ^🟋^27 | 1 | 15 | 14 | 0 | 6 | ^🟋^51 | 2 | 27 |
| **AK** | 20 | 1 | 22 | ^🟋^11 | 1 | 7 | ^🟋^40 | 2 | 38 |
| **TN** | 26 | 1 | 16 | 15 | 0 | 5 | 48 | 3 | 29 |
| **IMI** | 4 | 1 | 38 | 1 | 1 | 18 | 11 | 3 | 66 |
| **NI** | 10 | 0 | 13 | 8 | 0 | 12 | ^🟋^24 | 0 | 56 |
| **CO** | 11 | 0 | 32 | 5 | 0 | 15 | 29 | 0 | 51 |
| **C** | 4 | 0 | 38 | 1 | 0 | 19 | 9 | 0 | 71 |
| R: Resisitance, S: Sensetive, I: Intermediate; NA: Nalidixic acid, CIP: Ciprofloxacin, NOR: Norfloxacin, LEV: Levofloxacin, GAT: Gatifloxacin, OFX: Ofloxacin, CTX: Cefotaxime, CAZ: Ceftazidime, CRO: Ceftriaxone, ATM: Aztreonam, GM: Gentamicin, AK: Amikacin, TN: Tobramycin, IMI: Imipenem, NI: Nitrofurantoin, CO: Colistin, C: Chloramphenicol  ^🟋^: Significant | | | | | | | | | |
